# The lipid lowering effect of plant sterol ester capsules in hypercholesterolemic subjects

**DOI:** 10.1186/1476-511X-6-11

**Published:** 2007-04-09

**Authors:** Robert V Acuff, David J Cai, Zhi-Ping Dong, Doris Bell

**Affiliations:** 1 East Tennessee State University, College of Medicine, Johnson City, TN, USA; 2 Cognis Corporation, LaGrange, IL, USA; 3 Cognis Deutschland GmbH & Co. KG, Monheim, Germany

## Abstract

**Background:**

Foods enriched with phytosterols have been proven to be an effective therapy to improve blood lipid profiles. However, none of the studies have investigated the efficacy in lipid lowering of plant sterol esters (PSE) in capsule form. The objective of this study is to determine if the plant sterol esters (PSE) in capsule form (1.3 grams of PSE/day) lowered plasma cholesterol levels and lipid ratios in free-living hypercholesterolemic subjects during a 4-week intervention period.

**Methods:**

Sixteen subjects participated in a double-blind, placebo-controlled, sequential study with a 4-week placebo phase followed by a 2-week wash-out period and a 4-week treatment phase. Subjects were instructed to maintain stable diet pattern and physical activities. Blood samples were collected at 7, 21 and 28 days of each phase. The primary measurements were change in plasma total cholesterol (TC), HDL-cholesterol (HDL) and LDL-cholesterol (LDL) between phases and within each phase. The secondary measurements were change in triglycerides, lipoprotein ratios (TC/HDL, LDL/HDL) and C-reactive protein (CRP).

**Results:**

In comparison to placebo, LDL-cholesterol was significantly reduced by 7% and 4% (P < 0.05) at both week 3 and week 4; HDL at week 3 of the treatment was significantly increased by 9% (P < 0.01), but not at week 4 (4%); total cholesterol was not significantly different from placebo throughout the period, TC/HDL and LDL/HDL were significantly reduced by (8%, 8%, 6%, 10%, respectively) (P < 0.01) at both week 3 and week 4. CRP and triglycerides did not differ either between the two phases or during the treatment phase.

**Conclusion:**

In conclusion, plant sterol ester capsule is effective in improving lipid profiles among hypercholesterolemic subjects in a free-living setting at the minimum dosage recommended by FDA. The significant improved lipid profiles were reached after three weeks of administration. To achieve better lipid lowering results, higher dosages and combination with diets low in saturated fat and cholesterol are recommended.

## Background

Elevated LDL-cholesterol is a significant risk factor for coronary artery disease. The use of statin drugs is the current therapeutic option for lowering LDL-cholesterol and improving lipid profile in hypercholesteremic patients. According to the American Heart Association guidelines, other options should be considered as well to treat or prevent hypercholesterolemia, including the use of phytosterols. Phytosterols, either as plant sterols or plant stanols, are natural cholesterol-like substances derived from plants [1]. The main mechanism by which phytosterols reduce blood cholesterol is to inhibit cholesterol absorption in the small intestine. Therefore, the physical forms, carriers and solubilization of the phytosterols are important characteristics to determine the efficacy of phytosterols on cholesterol lowering [2].

In terms of physical forms, free phytosterols are water and oil insoluble. The efficacy on lowering blood cholesterol of free phytosterols is often dependant on the dispersion capability in water and oil [3-5]. Recently, soy lecithin has been used to form more dispersible complexes with free sterol or stanol resulting in more bioavailable free sterol/stanol formulation than previous formulations [6-8]. However, the improved efficacy has only been confirmed in tablet forms, but not in the capsule form [7,8]. Fatty acid esters of sterols or stanols, on the other hand, are oil soluble. Thus, they are more easily dispersible in oils than free sterols or stanols which make them a better choice for soft gel capsules than the free sterol/stanol.

In terms of carriers, there is abundant evidence suggesting LDL-cholesterol lowering efficacy of phytosterols either as plant sterols or stanols in food forms, including water emulsions [3]; water as lecithin micelles [9]; yogurt [10,11]; low fat milk [12,13]; chocolate [14]; cereal; snack bars, breads, and beverages [15,16]. However, there are very few studies that investigated if these compounds provided as pharmaceutical forms, such as tablets and capsules, offer the same benefits [2,7,8]. The information on non-food forms is essential for long term supplementation strategy. As indicated by Law et al, (1994 and 2003), to achieve life saving benefits from mortality associated with heart disease and stroke, the LDL-cholesterol lowering strategy needs to be maintained for at least two years, and preferably for five years [17,18]. The pharmaceutical dosage forms, such as tablets and capsules, can provide more convenience and flexibility needed for the recommended long term usage than the traditional food applications [2]. Furthermore, these forms could be better delivery vehicles for phytosterols to be incorporated into the combination therapeutic strategy with pharmaceutical agents to provide an additional LDL-cholesterol lowering effect [19-21]. Unfortunately, none of the studies with these pharmaceutical forms used plant sterol esters [2,8]. Considering plant sterol ester is a more dispersible form in oil than free phytosterols, phytosterol esters may be more suitable choice for soft gel capsules than free plant sterols/stanols.

The present study was designed to confirm the efficacy of the plant sterol esters (PSE) in capsule form on lipid profile lowering in free-living hypercholesterolemic subjects during a short term (4 weeks). The dosage used in the study, 1.3 g plant sterol ester (0.8 g free sterol equivalent), is the minimum dosage that is recommended by FDA's health claim [22]. The information will be essential for usage of plant sterol esters in supplement forms beyond current food applications.

## Results

No subject was dismissed because of the inability to tolerate the treatment or placebo or because of an adverse action or event. The statistical evaluation was determined based upon the 16 remaining subjects who completed the total study.

### Placebo vs. treatment

**Figure 1:**
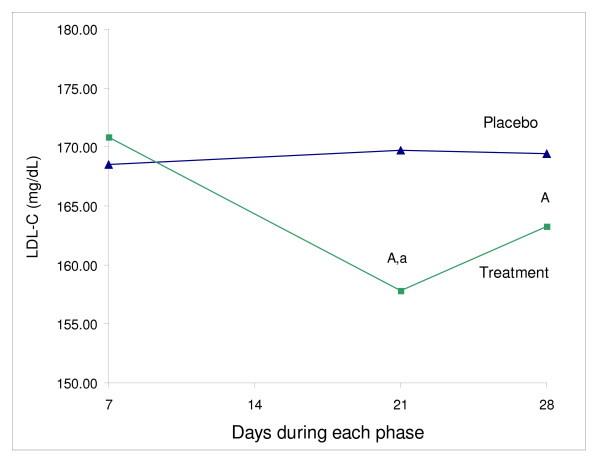
**The effect of plant sterol ester on LDL – cholesterol (mg/dL) (N = 16)**. ^A ^Significantly different from placebo, p < 0.05 (paired t-test) ^a ^Significantly different within phase, p < 0.05 (1-way repeated measure ANOVA)

**Figure 2:**
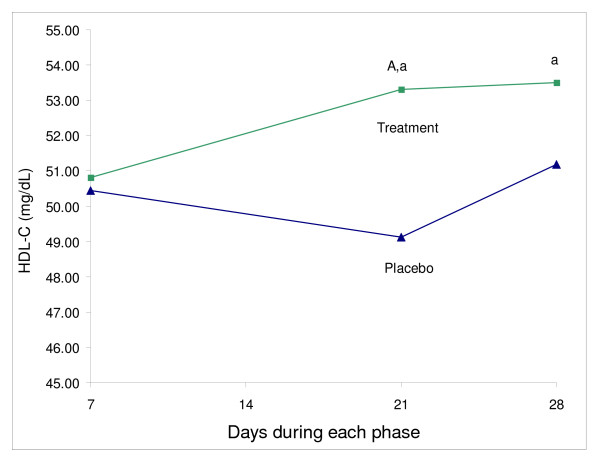
**The effect of plant sterol ester on HDL – cholesterol (mg/dL) (N = 16)**. ^A ^Significantly different from placebo, p < 0.05 (paired t-test) ^a ^Significantly different within phase, p < 0.05 (1-way repeated measure ANOVA)

Total cholesterol was reduced, but not significantly, at the end of treatment period (5%) (P = 0.07) (Table 1, Figure 1). LDL-cholesterol, TC/HDL, LDL/HDL ratios were significantly reduced by 7% (P < 0.05), 8% (P < 0.01) and 6% (P < 0.01) at day 21, 4% (P < 0.05), 8% (P < 0.01) and 10% (P < 0.01) at day 28, respectively (Table 1, Figure 1). HDL at day 21 of the treatment was significantly higher in comparison to placebo (9%, P < 0.01), but not at day 28 (4%) (Table 1, Figure 2). CRP and triglycerides did not differ between placebo and treatment phase.

**Table 1:** Effects of plant sterol ester capsules on plasma lipid profiles and CRP (n = 16)^1^

**Parameters**	**Initial Baseline **^2^	**Day 7**	**Day 21**	**Δ PSE-Placebo**	**Day 28**	**Δ PSE-Placebo**
**Total Cholesterol (mg/dL)**	256.0 ± 24.3					
Placebo phase		238.4 ± 18.8	239.3 ± 28.3		241.6 ± 27.2	
Treatment phase		239.9 ± 29.6	237.4 ± 29.2	-1.9 (-1%)	230.4 ± 22.3	-11.2 (-5%)
**LDL (mg/dL)**	177.1 ± 22.8					
Placebo phase		168.5 ± 22.9	169.8 ± 25.6		169.4 ± 27.0	
Treatment phase		170.8 ± 27.7	157.8 ± 22.8 ^A, a^	-12 (-7%)	163.3 ± 27.0^A^	-6.1 (-4%)
**HDL (mg/dL)**	57.8 ± 19.9					
Placebo phase		50.4 ± 13.8	49.1 ± 13.6		51.2 ± 15.4	
Treatment phase		50.8 ± 15.5	53.3 ± 16.3^A, a^	4.2 (9%)	53.5 ± 16.3^a^	2.3 (4%)
**Triglycerides**	125.9 ± 81.4					
Placebo phase		116.6 ± 60.8	122.7 ± 89.0		126.5 ± 74.6	
Treatment phase		111.4 ± 69.8	121.7 ± 71.4	-1 (< -1%)	115.7 ± 63.6	-10.8 (-9%)
**Total Cholesterol/HDL**	4.89 ± 1.64					
Placebo phase		5.1 ± 1.4	5.2 ± 1.6		5.1 ± 1.6	
Treatment phase		5.1 ± 1.5	4.8 ± 1.6^A, a^	-0.4 (-8%)	4.7 ± 1.4^A, a^	-0.4 (-8%)
**LDL/HDL**	3.44 ± 1.28					
Placebo phase		3.6 ± 1.3	3.6 ± 1.2		3.7 ± 1.2	
Treatment phase		3.7 ± 1.2	3.4 ± 1.2 ^A, a^	-0.2 (-6%)	3.3 ± 1.2^A, a^	-0.4 (-10%)
**CRP (mg/dL)**	0.8 ± 0.5					
Placebo phase		0.7 ± 0.4	0.7 ± 0.4		0.7 ± 0.5	
Treatment phase		0.8 ± 0.6	0.8 ± 0.5	0.07 (5%)	0.7 ± 0.3	0

^1 ^Values are means ± SD.

^2 ^Initial baseline measurement was done 6 week before the placebo phase. Therefore, the data was not included in any analysis, but are provided as reference information

^A ^Significantly different from placebo, p < 0.05 (paired t-test)

^a ^Significantly different within phase, p < 0.05 (1-way repeated measure ANOVA)

### Within each phase

There was no significant change in any of the measurements within the placebo phase (Table 1). Within the treatment phase, in comparison to day 7, LDL was significantly reduced at day 21(P < 0.05), but not at day 28 (Table 1, Figure 1); HDL was significantly increased at both day 21 and 28 (P < 0.05) (Table 1, Figure 2); TC/HDL and LDL/HDL ratios were significantly reduced at both day 21 and 28 (P < 0.001) (Table 1). Total cholesterol was reduced throughout the treatment phase, but this reduction was not significant. CRP and triglycerides did not change during the treatment phase.

## Discussion

The cholesterol lowering effect of plant sterols, either as free or esterified forms, is well documented in the literature. Most studies used food forms as delivery vehicles. There are only a few studies that have evaluated the effectiveness of phytosterols in tablet or capsule form, all of which used free stanols [7,8,23]. To our knowledge, the present study is the first study to demonstrate a significant reduction in plasma LDL cholesterol by 4% with plant sterol esters in capsules (P < 0.05). Epidemiological studies have shown that such a 4–5% reduction of LDL correlates with a 5–10% reduction in CHD risk in the first 5 years, and by 10% over a life time [24]. This result indicates that regular use of plant sterol esters in a soft gel capsule could contribute significant benefits to long term cholesterol management as the food delivery forms. Previous studies with phytosterol fortified foods have shown reductions of 5% or more in LDL cholesterol levels relative to a control. For example, in men and women with a wide range of age and baseline cholesterol levels, 0.8 g of free sterol equivalents administrated as sterol ester in spreads decreased LDL cholesterol by 6% [25], and 1.1 g/d of free sterol equivalents in spreads as plant sterol ester decreased LDL-cholesterol by 4.9% [26]. The significant 4% reduction in plasma LDL-C in the present study indicates that regular use of plant sterol esters is equally effective in a soft gel capsule form compared to food delivery forms of similar doses.

This study was conducted in free-living subjects without collection of dietary records at baseline or during the study. Subjects were advised to maintain regular dietary habits and physical activity. This practical approach was meant to mimic plant sterol capsules consumption under various background diets. Previously, plant sterols were said to be more effective when consumed with diets containing higher levels of cholesterol or fat [2,27], more recent studies indicated that since plant sterols impair both dietary and bilary cholesterol absorption, they are effective even when consumed with low fat diets [28-30]. Thus, the delivery regimen for the plant sterols is more crucial than the background diet. In the present study, all subjects were advised to take the capsules with each meal (lunch and dinner). As long as plant sterols are taken with meals to stimulate the bilary flow, they can effectively lower cholesterol within the context of various diets and food forms [2].

The present study showed significantly (p < 0.01) improved lipid ratios (TC/HDL and LDL/HDL) either independent of placebo or relative to placebo. These results are in agreement with previous reported studies using free stanols [7,8] or sterol esters [31]. The improved lipid ratios are due to reduced LDL, total cholesterol and increased HDL levels during the treatment phase. While most studies published to date have reported that phytosterols have little or no effect on HDL, even with long-term use [5,32], in our study HDL level was increased during the treatment period as compared to placebo (P < 0.05). A recent study using orange juice with plant sterols also observed an increase in HDL [33], In studies using plant sterol esters in combination with exercise, significant increase in HDL was observed [34,35]. Since this was a cross-over study and the effect was not observed during placebo phase, it is likely to be a consequence of plant sterol ester. A longer term administration is needed to further confirm the positive effect of plant sterol ester on HDL levels.

Due to the difference in physical and chemical characteristics between esterized and free forms, plant sterol esters may be a better choice for capsules than their free form. Theoretically, both esters and free forms should possess similar cholesterol lowering effect if the free sterols are properly solubilized. Improperly dispersed formulations, such as aqueous or oil-based suspensions of solid sterols, have been reported as unsuccessful in lowering LDL-cholesterol. These formulations included a crystalline aqueous suspension of sitosterol [36], capsules containing 3 g of stanol powder dispersed in safflower oil [23], or free sterols in beverages with low fat [6]. In addition to the dispersion factor, these studies suggested that the crystal form of free sterol/stanols may be another important factor determining their solubility in intestinal fluid and their effectiveness to inhibit cholesterol absorption [37]. To enhance its solubilization in water or oils, the free plant sterols or stanols are usually added to other substances and often require the presence of fat to facilitate the emulsion. Thus, the method of dispersion, processing, and use of emulsifiers, surfactants, and crystal habit modifiers plays a crucial role in delivering small crystals of free phytosterols over time to sufficiently maintain their bioavailability [2,12,13]. A recent breakthrough in formulation, using soy lecithin to form more water dispersible and bioavailable complexes with free stanol, has been demonstrated to lower plasma cholesterol and LDL in two recent studies in tablet form [7,8]. In a placebo controlled, double-blind study by McPherson et al. [7], free stanols were emulsified with soy lecithin and the spray dried preparation was used to make tablet and capsule forms that were tested in 26 subjects per group over a 6-week period. The group that received tablet form experienced reduction in both LDL cholesterol and LDL/HDL ratio by 10.4% and 11.5% respectively. However, no reduction was observed in the group that received capsules [7]. The author attributed the difference in LDL reduction to the variation in disintegration times, in so far that the tablet disintegrated much faster than the capsule form (10 min. vs. 45 min., respectively).

Fatty acid esters of sterols or stanols are oil soluble. Thus, they are more easily dispersible in oils than free sterols or stanols. Additionally, plant sterol esters are well distributed in the small intestine providing ample surface area for incorporation into bile salt micelles through the digestive process in order to inhibit cholesterol absorption [2,24]. Earlier evidence suggested that even though the cholesterol lowering effect is similar between free sterols and stanols, the saturation state of their esterified forms may affect the ability to reduce cholesterol absorption [38]. In a clinical study conducted by Jones et al. (2000), intake of plant sterol esters from margarine resulted in larger LDL reductions than margarine with stanol ester (13% vs. 8%, respectively) [38]. However, taking the totality of evidence into account, sterol and stanol esters are probably equal in their cholesterol-lowering effect. Nevertheless, plant sterol esters remain a better choice for soft gel capsules despite the improvements in free sterols formulation to increase the bioavailability.

Finally, this study evaluated the effect of PSE on CRP, a proposed risk factor in cardiovascular disease [39]. All subjects in this study had low or average CRP levels (<3.0 mg/L) at baseline and PSE had no effect on CRP during the treatment phase. It has been demonstrated that diet can have a beneficial impact on CRP levels as observed in the "portfolio diet", a dietary prescription comprised of several foods and food components (including plant sterol esters) which are known to reduce cholesterol levels [40]. In the present study, due to the free-living study design, it is unlikely that CRP will be changed solely based on plant sterol ester treatment.

There are several limitations of this study. First, the study treatments were not randomized. The placebo and PSE treatment were conducted sequentially with wash-out period in between. There is some evidence suggesting a carry-over effect from phytosterol treatment to the placebo group [41] in randomized crossover design. The sequential design could minimize the carry-over effect since the placebo phase was performed first. To minimize the potential confounding effect due to the sequential design, such as time drifting effect, additional within group comparisons were conducted using repeated measure analysis. The results from placebo phase did not show any significant change within the 4-week period whereas significant changes were found within the treatment phase. This within group analyses confirmed that the significant changes were due to the treatment, not the time drifting effect.

Another limitation is the lack of true baseline measurements at the beginning of placebo and treatment phase. This was not intended for the original study design, but occurred during the study. To compensate for this limiting factor, the within group analyses were performed to compare measurements at day 21 and 28 to day 7 of each phase. The significant differences were only detected during the treatment phase, not the placebo phase. Additionally, there was no significant difference at day 7 of each phase between the placebo and treatment suggesting a significant treatment effect.

The number of subjects was calculated based on 10% reduction of total cholesterol. However, given the fact that the tested dose was 1.3 g plant sterol esters/day as 0.8 g free sterol equivalent/day, it is arguable that the above reduction is overestimated. Thus, the study may be underpowered which could explain the lack of significance in reduction of total cholesterol (-5%) (P = 0.07). More subjects are needed for further studies with the above dosage. Nevertheless, the present study showed a significant improvement in LDL-C occurred with the present number of subjects indicating that the effect of plant sterol ester is significant.

## Conclusion

This study is the first study that confirmed regular use of plant sterol ester capsules is an effective strategy in improving lipid profiles, especially lipoprotein ratios, among hypercholesterolemic subjects in a free-living setting without dietary intervention. The clinical advantage of the capsule form is that it provides a convenient vehicle to consume without the dietary impact on calories. It can also easily be incorporated into a cholesterol-lowering regimen in standard clinical practice with counseling or therapeutic lifestyle changes including those recommended by the National Cholesterol Education Program [24,42]. The significant improvement in LDL-C occurred in free-living subjects with little dietary control indicates that the effect of plant sterol ester is fairly significant regardless of the delivery vehicle and background diet. However, to achieve better lipid lowering effects, higher dosages and incorporation of cholesterol-lowering regimen are recommended. Further studies are needed to confirm the long term benefit of plant sterol ester capsules on blood cholesterol in populations with varying baseline cholesterol levels.

## Methods

### Subjects

The research protocol and informed consent were approved by the East Tennessee State University (ETSU) Institutional Review Board (IRB) Committee on Protection of Human Subjects.

Subject recruitment was conducted by campus advertisement. The questionnaire evaluation and hematological screening of potential subjects were conducted at the Nutrition Center in the Department of Internal Medicine. Inclusion criteria included men and women (not pregnant or breast feeding), borderline high or elevated serum lipid parameters, body mass index (BMI) < 30, ability to maintain stable diet and physical activity, and in otherwise healthy condition. Candidates were excluded from the study if they had

• diseases or conditions requiring drug intervention;

• been taking cholesterol lowering drugs, supplements or other practices, such as taking cloves of garlic every day

• uncontrolled elevated blood pressure (systolic >160 mmHg or diastolic > 95 mmHg)

• history or presence of drug or alcohol abuse, or alcohol intake >7 alcoholic beverages per week within the past 4 weeks

Power calculations based on expected 10% changes in total cholesterol levels determined that at least 16 subjects would be needed. Twenty subjects (total cholesterol 227–308 mg/dL, LDL 129–211 mg/dL and triglycerides 43–355 mg/dL) were included in the study. During the study phases, four subjects later withdrew due to either personal reasons or unable following restrictions, such as not taking cholesterol lowering supplements. The remaining sixteen subjects (12 female/4 male, age: 51 ± 13) (total cholesterol: 256 ± 24 mg/dL; LDL: 177 ± 23 mg/dL) completed the study. Table 2 presents the baseline means for the parameters of interest, including age and lipid values, for the 16 subjects who completed the study. Only data from the sixteen subjects were included for analysis.

**Table 2:** Subject Characteristics at Baseline (n = 16)

Variable	Mean + SD	Range
Age (yrs)	51 ± 13	25–72
Number of Subjects: Female/Male	12/4	
Total Cholesterol (mg/dL)	256.0 ± 24.3	227–308
LDL-Cholesterol (mg/dL)	177.1 ± 22.8	129–211
HDL-Cholesterol (mg/dL)	57.8 ± 19.9	36–110
Triglycerides (mg/dL)	125.9 ± 81.4	43–355
Total Cholesterol/HDL	4.89 ± 1.64	2.25–8.11
LDL-Cholesterol/HDL	3.44 ± 1.28	1.17–5.69
C-Reactive Protein (mg/dL)	0.8 ± 0.5	0.5–1.9

* SD = Standard Deviation

### Materials

Both placebo (containing soybean oil) and matching plant sterol ester capsules were manufactured by Cardinal Health (Dublin, OH). The plant sterol esters (Vegapure^® ^95) were manufactured by Cognis Corporation (LaGrange, IL). The plant sterol esters were prepared by esterification of free plant sterols from a mixture of soy, rapeseed and other vegetable sources, with fatty acids from sunflower oil. Mixed tocopherols and ascorbyl palmitate are used in the formulation as antioxidants and chemical preservatives. Soybean oil is used as a coating agent and texturizer (see Table 3, Table 4).

**Table 3:** Composition of Vegapure^® ^95

**Component**	**Amount (% W/W)**
Mixed Phytosterol Esters and Phytosterols	Min. 97.0%
Ascorbyl Palmitate	250 ppm
Mixed Tocopherols	180 ppm
Soybean Oil	70 ppm
Total	100.0%

**Table 4:** Sterol composition of Vegapure^® ^95 (as free sterols)

**Component**	**Average Percent**
β-Sitosterol	49
Campesterol	24
Stigmasterol	17
Brassicasterol	4
Campestanol	3
Sitostanol	1
Other sterols	2

### Study design and intervention phases

The study was a double-blind, placebo-controlled, non-randomized, sequential study. After the initial baseline measurements which were conducted in late December, there was a six-week period before the placebo phase to minimize lipid profile variance. For this reason, the initial baseline data was not used for comparison to any data collected in placebo and treatment phases

Six weeks after the initial baseline measurement, all subjects were assigned to a placebo phase (soybean oil) for four weeks followed by a two-week, wash-out period before proceeding to a four-week treatment phase. Subjects were instructed to take 1 capsule at each of 2 meals per day (lunch and dinner) during each phase. The PSE treatment provided a total of 1.3 grams per day of sterol esters (0.8 grams free plant sterol equivalents). This amount is the minimum dose recommended in FDA's health claim for phytosterols [22].

At the beginning of each phase, each subject was given a bottle containing 60 capsules (56 capsules are needed for 4 weeks and 4 capsules are extra). Placebo capsules and plant sterol ester capsules were identical in terms of appearance and sensory characteristics. Compliance was monitored by counting extra capsules in the bottles at the end of each phase and questioning subjects regarding missed doses. Subjects were instructed to maintain their stable diet pattern and physical activity level during the study periods. Although general advice on healthy life style was given to subjects occasionally during their visit to the Nutrition Center, neither dietary advice was given, nor were food records collected.

Fasted blood samples were collected at 7, 21 and 28 days of each phase. The primary measurements were change of plasma total cholesterol (TC), HDL-cholesterol and LDL-cholesterol between placebo and treatment. The secondary measurements were changes of triglycerides, lipoprotein ratios (TC/HDL, LDL/HDL) and CRP.

### Lipids and C-Reactive Protein (CRP) analyses

Fasted serum samples were analyzed for triglycerides (TG), total cholesterol (TC), high density lipoprotein cholesterol (HDL-C), low density lipoprotein cholesterol (LDL-C), and c-reactive protein (CRP). Total cholesterol, HDL, triglycerides and CRP were performed at Johnson City Medical Center Hospital (Johnson City, TN) using an automated clinical chemistry analyzer (Beckman Synchron LX20; Fullerton, CA). LDL-cholesterol was calculated using the Friedewald formula.

### Statistical analysis

Data were analyzed to detect significant differences between placebo and treatment by ANOVA. Once the significant difference was detected, paired student t-tests was performed to compare the measurements at each blood collection time (7 day, 21 day and 28 day) between placebo and treatment group. Additional within-group analysis was performed by 1-way repeated measures analysis of ANOVA to compare measurements within placebo or treatment phase in order to make sure that the expected changes were not caused by time drift during each phase. Paired student t-tests were performed to compare measurements at each blood collection time.

## Abbreviations

BMI = body mass index

CHD = coronary heart disease

CRP = c-reactive protein

dL = deciliter

ETSU = East Tennessee State University

FDA = Food and Drug Administration

g = gram

GCP = Good Clinical Practice

GLP = Good Laboratory Practice

GRAS = Generally Recognized as Safe

HDLC = high-density lipoprotein cholesterol

HPLC = High Performance Liquid Chromatography

Hg = mercury

IRB = Institutional Review Board

L = liter

LDLC = low density lipoprotein cholesterol

mg = milligram

NCEP = National Cholesterol Education Program

PSE = plant sterol esters

TC = total cholesterol

TG = triglycerides

## Competing interests

All authors have read and approved this manuscript. RVA and ZPD do not have competing interests from the sponsor. DJC and DB are research scientists at Cognis Corp. (USA) and Cognis Deutschland GmbH & Co. KG, respectively, who were involved in interpreting the data and preparing the manuscript, but were not involved in conducting the study.

## Authors' contributions

RVA established the study design, obtained study funding and contributed to data interpretation. ZPD recruited subjects, completed instrumental analysis and data collection. DJC wrote the manuscript, conducted statistical analysis, contributed to data interpretation and final revision. DB contributed to data interpretation and revised the manuscript.
